# Correction: Impacts of a sugar sweetened beverage tax on body mass index and obesity in Thailand: A modelling study

**DOI:** 10.1371/journal.pone.0257438

**Published:** 2021-09-10

**Authors:** Payao Phonsuk, Vuthiphan Vongmongkol, Suladda Pongutta, Rapeepong Suphanchaimat, Nipa Rojroongwasinkul, Boyd Anthony Swinburn

The third author’s name is misspelled. The correct spelling is: Suladda Pongutta. The correct citation is: Phonsuk P, Vongmongkol V, Pongutta S, Suphanchaimat R, Rojroongwasinkul N, Swinburn BA (2021) Impacts of a sugar sweetened beverage tax on body mass index and obesity in Thailand: A modelling study. PLoS ONE 16(4): e0250841. https://doi.org/10.1371/journal.pone.0250841

Additionally, the author’s name is misspelled in Reference 30. The correct citation is: Pongutta S, Chongwatpol P, Tantayapirak P, Vandevijvere S. Declaration of nutrition information on and nutritional quality of Thai ready-to-eat packaged food products. Public Health Nutrition. 2018;21(8):1409–17. https://doi.org/10.1017/s1368980017003792.
