# Supplementary material for: Impacts of a sugar sweetened beverage tax on body mass index and obesity in Thailand: A modelling study
Source: PLoS One. 2021 Apr 29;16(4):e0250841. doi: 10.1371/journal.pone.0250841 (PMC8084227; doi:10.1371/journal.pone.0250841)
Supplement: S1 Table — Baseline data of (a) daily SSB consumption in ml/day/person and (b) energy intake in kJ/person/day by sex and age groups among Thais. (DOCX) [file pone.0250841.s001.docx]

# Supporting information

**S1 Table.** **Baseline data of (a) daily SSB consumption in ml/day/person and (b) energy intake in kJ/person/day by sex and age groups among Thais**

**(a) Daily SSB consumption in ml/day/person by sex and age groups**

| **Gender** | **Age groups (years)** | **Number of samples** | **SSB consumption, ml/person/day**  **Mean (95% CI)** | **Standard error** |
| --- | --- | --- | --- | --- |
| **Men** | **3-5** | 620 | 149.1  (133.0, 165.2) | 8.2 |
|  | **6-12** | 606 | 227.2  (209.2, 245.2) | 9.2 |
|  | **13-17** | 489 | 378.4  (348.1, 408.6) | 15.4 |
|  | **18-34** | 539 | 422.5  (387.3, 457.8) | 17.9 |
|  | **35-64** | 561 | 197.5  (175.9, 219.2) | 11.0 |
|  | **65 or older** | 576 | 72.7  (60.8, 84.7) | 6.1 |
|  | **All men** | **3,391** | **234.6**  **(224.5, 244.7)** | **5.1** |
| **Women** | **3-5** | 565 | 133.4  (118.7, 148.1) | 7.5 |
|  | **6-12** | 633 | 222.2  (200.8, 243.6) | 10.9 |
|  | **13-17** | 493 | 293.2  (268.7, 317.8) | 12.5 |
|  | **18-34** | 621 | 291.8  (266.1, 317.6) | 13.1 |
|  | **35-64** | 725 | 123.0  (107.6, 138.4) | 7.8 |
|  | **65 or older** | 570 | 35.1  (28.7, 41.5) | 3.3 |
|  | **All women** | **3,607** | **180.5**  **(172.1, 188.8)** | **4.2** |
| **All** | | **6,998** | **206.7**  **(200.2, 213.3)** | **3.3** |

**(b) Energy intake in kJ/person/day by sex and age groups**

| **Gender** | **Age groups (years)** | **Number of samples** | **Energy intake,**  **kJ/person/day**  **Mean (95% CI)** | **Standard Error** |
| --- | --- | --- | --- | --- |
| **Men** | **3-5** | 620 | 304.1  (271.2, 336.9) | 16.7 |
|  | **6-12** | 606 | 463.5  (426.8, 500.2) | 18.7 |
|  | **13-17** | 489 | 771.9  (710.2, 833.5) | 31.4 |
|  | **18-34** | 539 | 861.9  (790.1, 933.8) | 36.6 |
|  | **35-64** | 561 | 403.0  (358.8, 447.1) | 22.5 |
|  | **65 or older** | 576 | 148.4  (124.1, 172.7) | 12.4 |
|  | **All men** | **3,391** | **478.6**  **(458.0, 499.2)** | **10.5** |
| **Women** | **3-5** | **565** | 272.2  (242.2, 302.1) | **15.3** |
|  | **6-12** | 633 | 453.3  (409.6, 497.0) | 22.3 |
|  | **13-17** | 493 | 598.2  (548.2, 648.2) | 25.5 |
|  | **18-34** | 621 | 595.3  (542.8, 647.8) | 26.8 |
|  | **35-64** | 725 | 250.9  (219.5, 282.3) | 16.0 |
|  | **65 or older** | 570 | 71.6  (58.5, 84.7) | 6.7 |
|  | **All women** | **3,607** | **368.2**  **(351.1, 385.2)** | **8.7** |
| **All** | | **6,998** | **421.7**  **(408.3, 435.1)** | **6.8** |

DOI: 10.6084/m9.figshare.14256296
