# Supplementary material for: Impacts of a sugar sweetened beverage tax on body mass index and obesity in Thailand: A modelling study
Source: PLoS One. 2021 Apr 29;16(4):e0250841. doi: 10.1371/journal.pone.0250841 (PMC8084227; doi:10.1371/journal.pone.0250841)
Supplement: S2 Table — Baseline data of (a) weight (kg) and (b) body mass index (BMI) by sex and age groups among Thais. (DOCX) [file pone.0250841.s002.docx]

# Supporting information

**S2 Table.** **Baseline data of (a) weight (kg) and (b) body mass index (BMI) by sex and age groups among Thais**

**(a) Weight (kg) by sex and age groups**

| **Gender** | **Age groups (years)** | **Number of samples** | **Weight, kg**  **Mean (95% CI)** | **Standard error** |
| --- | --- | --- | --- | --- |
| **Men** | **3-5** | 620 | 17.4  (17.0, 17.7) | 0.2 |
|  | **6-12** | 606 | 31.4  (30.6, 32.2) | 0.4 |
|  | **13-17** | 489 | 51.0  (50.2, 51.9) | 0.4 |
|  | **18-34** | 539 | 68.3  (66.9, 69.7) | 0.7 |
|  | **35-64** | 561 | 65.8  (64.7, 66.8) | 0.5 |
|  | **65 or older** | 576 | 60.4  (59.5, 61.4) | 0.5 |
|  | **All men** | **3,391** | **48.1**  **(47.4, 48.9)** | **0.4** |
| **Women** | **3-5** | 565 | 17.1  (16.8, 17.4) | 0.2 |
|  | **6-12** | 633 | 32.2  (31.4, 33.0) | 0.4 |
|  | **13-17** | 493 | 47.7  (47.0, 48.3) | 0.3 |
|  | **18-34** | 621 | 58.7  (57.5, 59.9) | 0.6 |
|  | **35-64** | 725 | 62.0  (61.2, 62.9) | 0.4 |
|  | **65 or older** | 570 | 53.0  (52.2, 53.9) | 0.5 |
|  | **All women** | **3,607** | **45.7**  **(45.2, 46.4)** | **0.3** |
| **All** | | **6,998** | **46.9**  **(46.4, 47.4)** | **0.3** |

**(b) Body mass index (BMI) by sex and age groups**

| **Gender** | **Age groups (years)** | **Number of samples** | **BMI, kg/m2**  **Mean (95% CI)** | **Standard Error** |
| --- | --- | --- | --- | --- |
| **Men** | **3-5** | 620 | 15.7  (15.6, 15.9) | 0.1 |
|  | **6-12** | 606 | 17.1  (16.8, 17.3) | 0.1 |
|  | **13-17** | 489 | 18.7  (18.5, 19.0) | 0.1 |
|  | **18-34** | 539 | 23.8  (23.3, 24.2) | 0.2 |
|  | **35-64** | 561 | 24.2  (23.9, 24.6) | 0.2 |
|  | **65 or older** | 576 | 23.1  (22.8, 23.5) | 0.2 |
|  | **All men** | **3,391** | **20.3**  **(20.2, 20.5)** | **0.1** |
| **Women** | **3-5** | 565 | 15.5  (15.3, 15.8) | 0.1 |
|  | **6-12** | 633 | 17.0  (16.7, 17.2) | 0.1 |
|  | **13-17** | 493 | 19.4  (19.2, 19.6) | 0.1 |
|  | **18-34** | 621 | 23.7  (23.2, 24.1) | 0.2 |
|  | **35-64** | 725 | 26.2  (25.8, 26.5) | 0.2 |
|  | **65 or older** | 570 | 23.6  (23.3, 24.0) | 0.2 |
|  | **All women** | **3,607** | **21.1**  **(21.0, 21.3)** | **0.1** |
| **All** | | **6,998** | **20.8**  **(20.6, 20.9)** | **0.1** |

DOI: 10.6084/m9.figshare.14256302
