# Supplementary material for: Impacts of a sugar sweetened beverage tax on body mass index and obesity in Thailand: A modelling study
Source: PLoS One. 2021 Apr 29;16(4):e0250841. doi: 10.1371/journal.pone.0250841 (PMC8084227; doi:10.1371/journal.pone.0250841)
Supplement: S3 Table — (DOCX) [file pone.0250841.s003.docx]

# Supporting information

**S3 Table.** **The differences of sex and age groups and change in consumption, change in weight and change in BMI in three different tax scenarios**

**a) Tax rate 11%**

|  | | **Coef.** |  | **Std. Err.** | **t** | **P-Value*** | **[95% Confidence Interval]** | |  |
| --- | --- | --- | --- | --- | --- | --- | --- | --- | --- |
|  |  |  |  |  |  |  | **Lower bound** | **Upper bound** |  |
|  | **1) Change in SSB consumption** | | | | | | | | |
|  | **Sex** (Male = reference) | | | | | | | | |
| Female | | 7.97 |  | 0.87 | 9.21 | 0.000 | 6.27 | 9.66 |  |
|  | **Age** **groups** (3-5.9 = reference) | | | | | | | | |
| 6- 12.9 | | -11.90 |  | 1.47 | -8.11 | 0.000 | -14.77 | -9.02 |  |
| 13- 17.9 | | -27.37 |  | 1.56 | -17.56 | 0.000 | -30.42 | -24.31 |  |
| 18- 34.9 | | -30.00 |  | 1.49 | -20.10 | 0.000 | -32.92 | -27.07 |  |
| 35- 64.9 | | -2.64 |  | 1.46 | -1.81 | 0.070 | -5.50 | 0.21 |  |
| >= 65 | | 12.10 |  | 1.50 | 8.09 | 0.000 | 9.16 | 15.03 |  |
|  | **2) Change in weight** | | | | | | | | |
|  | **Sex** (Male = reference) | | | | | | | | |
| Female | | 0.14 |  | 0.02 | 9.39 | 0.000 | 0.11 | 0.17 |  |
|  | **Age** **groups** (3-5.9 = reference) | | | | | | | | |
| 6- 12.9 | | -0.18 |  | 0.03 | -6.99 | 0.000 | -0.23 | -0.13 |  |
| 13- 17.9 | | -0.61 |  | 0.03 | -22.11 | 0.000 | -0.67 | -0.56 |  |
| 18- 34.9 | | -0.82 |  | 0.03 | -30.97 | 0.000 | -0.88 | -0.77 |  |
| 35- 64.9 | | -0.26 |  | 0.03 | -10.20 | 0.000 | -0.32 | -0.21 |  |
| >= 65 | | 0.03 |  | 0.03 | 1.31 | 0.190 | -0.02 | 0.09 |  |
|  | **3) Change in BMI** | | | | | | | | |
|  | **Sex** (Male = reference) | | | | | | | | |
| Female | | 0.04 |  | 0.01 | 5.78 | 0.000 | 0.02 | 0.05 |  |
|  | **Age** **groups** (3-5.9 = reference) | | | | | | | | |
| 6- 12.9 | | -0.03 |  | 0.01 | -2.35 | 0.019 | -0.05 | 0.00 |  |
| 13- 17.9 | | -0.14 |  | 0.01 | -11.65 | 0.000 | -0.16 | -0.11 |  |
| 18- 34.9 | | -0.20 |  | 0.01 | -18.21 | 0.000 | -0.23 | -0.18 |  |
| 35- 64.9 | | -0.00 |  | 0.01 | -0.16 | 0.871 | -0.02 | 0.02 |  |
| >= 65 | | 0.11 |  | 0.01 | 10.02 | 0.000 | 0.09 | 0.13 |  |

*Statistically significant P-value >0.0001

**b) Tax rate 20%**

|  | **Coef.** | **Std. Err.** | **t** | **P-Value*** | **[95% Confidence Interval]** | |  |
| --- | --- | --- | --- | --- | --- | --- | --- |
|  |  |  |  |  | **Lower bound** | **Upper bound** |  |
| **1) Change in SSB consumption** | | | | | | | |
| **Sex** (Male = reference) | | | | | | | |
| Female | 14.80 | 1.61 | 9.21 | 0.000 | 11.65 | 17.95 |  |
| **Age** **groups** (3-5.9 = reference) | | | | | | | |
| 6- 12.9 | -22.10 | 2.73 | -8.11 | 0.000 | -27.44 | -16.75 |  |
| 13- 17.9 | -50.82 | 2.89 | -17.56 | 0.000 | -56.49 | -45.15 |  |
| 18- 34.9 | -55.71 | 2.77 | -20.10 | 0.000 | -61.15 | -50.28 |  |
| 35- 64.9 | -4.91 | 2.70 | -1.81 | 0.070 | -10.21 | 0.39 |  |
| >= 65 | 22.47 | 2.78 | 8.09 | 0.000 | 17.02 | 27.91 |  |
| **2) Change in weight** | | | | | | | |
| **Sex** (Male = reference) | | | | | | | |
| Female | 0.27 | 0.03 | 9.39 | 0.000 | 0.21 | 0.32 |  |
| **Age** **groups** (3-5.9 = reference) | | | | | | | |
| 6- 12.9 | -0.34 | 0.05 | -6.99 | 0.000 | -0.43 | -0.24 |  |
| 13- 17.9 | -1.14 | 0.05 | -22.11 | 0.000 | -1.24 | -1.04 |  |
| 18- 34.9 | -1.53 | 0.05 | -30.97 | 0.000 | -1.63 | -1.43 |  |
| 35- 64.9 | -0.49 | 0.05 | -10.20 | 0.000 | -0.59 | -0.40 |  |
| >= 65 | 0.06 | 0.05 | 1.31 | 0.190 | -0.03 | 0.16 |  |
| **3) Change in BMI** | | | | | | | |
| **Sex** (Male = reference) | | | | | | | |
| Female | 0.07 | 0.01 | 5.78 | 0.000 | 0.05 | 0.09 |  |
| **Age** **groups** (3-5.9 = reference) | | | | | | | |
| 6- 12.9 | -0.05 | 0.02 | -2.35 | 0.019 | -0.09 | -0.01 |  |
| 13- 17.9 | -0.25 | 0.02 | -11.65 | 0.000 | -0.29 | -0.21 |  |
| 18- 34.9 | -0.38 | 0.02 | -18.21 | 0.000 | -0.42 | -0.34 |  |
| 35- 64.9 | -0.00 | 0.02 | -0.16 | 0.871 | -0.04 | 0.04 |  |
| >= 65 | 0.21 | 0.02 | 10.02 | 0.000 | 0.17 | 0.25 |  |

*Statistically significant P-value >0.0001

**c) Tax rate 25%**

|  | **Coef.** | **Std. Err.** | **t** | **P-Value*** | **[95% Confidence Interval]** | |  |
| --- | --- | --- | --- | --- | --- | --- | --- |
|  |  |  |  |  | **Lower bound** | **Upper bound** |  |
| **1) Change in SSB consumption** | | | | | | | |
| **Sex** (Male = reference) | | | | | | | |
| Female | 18.21 | 1.98 | 9.21 | 0.000 | 14.34 | 22.09 |  |
| **Age** **groups** (3-5.9 = reference) | | | | | | | |
| 6- 12.9 | -27.20 | 3.35 | -8.11 | 0.000 | -33.77 | -20.62 |  |
| 13- 17.9 | -62.55 | 3.56 | -17.56 | 0.000 | -69.53 | -55.57 |  |
| 18- 34.9 | -68.57 | 3.41 | -20.10 | 0.000 | -75.26 | -61.88 |  |
| 35- 64.9 | -6.04 | 3.33 | -1.81 | 0.070 | -12.56 | 0.49 |  |
| >= 65 | 27.65 | 3.42 | 8.09 | 0.000 | 20.95 | 34.35 |  |
| **2) Change in weight** | | | | | | | |
| **Sex** (Male = reference) | | | | | | | |
| Female | 0.33 | 0.04 | 9.39 | 0.000 | 0.26 | 0.40 |  |
| **Age** **groups** (3-5.9 = reference) | | | | | | | |
| 6- 12.9 | -0.42 | 0.06 | -6.99 | 0.000 | -0.53 | -0.30 |  |
| 13- 17.9 | -1.40 | 0.06 | -22.11 | 0.000 | -1.53 | -1.28 |  |
| 18- 34.9 | -1.88 | 0.06 | -30.97 | 0.000 | -2.00 | -1.76 |  |
| 35- 64.9 | -0.60 | 0.06 | -10.20 | 0.000 | -0.72 | -0.49 |  |
| >= 65 | 0.08 | 0.06 | 1.31 | 0.190 | -0.04 | 0.20 |  |
| **3) Change in BMI** | | | | | | | |
| **Sex** (Male = reference) | | | | | | | |
| Female | 0.09 | 0.01 | 5.78 | 0.000 | 0.06 | 0.11 |  |
| **Age** **groups** (3-5.9 = reference) | | | | | | | |
| 6- 12.9 | -0.06 | 0.03 | -2.35 | 0.019 | -0.11 | -0.01 |  |
| 13- 17.9 | -0.31 | 0.03 | -11.65 | 0.000 | -0.36 | -0.26 |  |
| 18- 34.9 | -0.46 | 0.03 | -18.21 | 0.000 | -0.51 | -0.41 |  |
| 35- 64.9 | -0.00 | 0.02 | -0.16 | 0.871 | -0.05 | 0.04 |  |
| >= 65 | 0.26 | 0.03 | 10.02 | 0.000 | 0.21 | 0.31 |  |

*Statistically significant P-value >0.0001

DOI: 10.6084/m9.figshare.14260631
