# Supplementary material for: Impacts of a sugar sweetened beverage tax on body mass index and obesity in Thailand: A modelling study
Source: PLoS One. 2021 Apr 29;16(4):e0250841. doi: 10.1371/journal.pone.0250841 (PMC8084227; doi:10.1371/journal.pone.0250841)
Supplement: Attachment — Submitted filename: Response to Reviewers.docx [file pone.0250841.s004.docx]

**Response to Reviewers**

Journal requirements:

When submitting your revision, we need you to address these additional requirements.

1. Please ensure that your manuscript meets PLOS ONE's style requirements, including those for file naming. The PLOS ONE style templates can be found at

https://journals.plos.org/plosone/s/file?id=wjVg/PLOSOne_formatting_sample_main_body.pdf and

https://journals.plos.org/plosone/s/file?id=ba62/PLOSOne_formatting_sample_title_authors_affiliations.pdf

***Response:*** *The authors have checked the criteria requirement*s *and have improved the draft accordingly*.

2. We note that you have indicated that data from this study are available upon request. PLOS only allows data to be available upon request if there are legal or ethical restrictions on sharing data publicly. For information on unacceptable data access restrictions, please see http://journals.plos.org/plosone/s/data-availability#loc-unacceptable-data-access-restrictions.

In your revised cover letter, please address the following prompts:

a) If there are ethical or legal restrictions on sharing a de-identified data set, please explain them in detail (e.g., data contain potentially identifying or sensitive patient information) and who has imposed them (e.g., an ethics committee). Please also provide contact information for a data access committee, ethics committee, or other institutional body to which data requests may be sent.

b) If there are no restrictions, please upload the minimal anonymized data set necessary to replicate your study findings as either Supporting Information files or to a stable, public repository and provide us with the relevant URLs, DOIs, or accession numbers. Please see http://www.bmj.com/content/340/bmj.c181.long for guidelines on how to de-identify and prepare clinical data for publication. For a list of acceptable repositories, please see http://journals.plos.org/plosone/s/data-availability#loc-recommended-repositories.

We will update your Data Availability statement on your behalf to reflect the information you provide.

***Response:*** *Concerning the availability of data used in this study, we have stated that data are available upon request. To elaborate on this, the data is owned by the National Bureau of Agricultural Commodity and Food Standards (ACFS), Thailand. We, thus, have officially asked for their permission to use the data in our study. However, we can provide the minimal data set to replicate study findings as in the supporting information (S1 Table and S2 Table). Furthermore, the DOIs for this data set have been created.*

3. Please ensure that you refer to Figures 1 and 2 in your text as, if accepted, production will need this reference to link the reader to the figure.

***Response:*** *The authors have mentioned Fig 1 and Fig 2 also the supporting information in the draft.*

Reviewer #1: In is an interesting article that could help in policies to reduce the prevalence of obesity. Overall, the manuscript is well written but need several improvements:

1. Results.

- Comparison were made between men and women as well as different age groups for all data but there have been no mention whether the differences were significant or not. There was a mention about differences in energy intake across age groups but no mention about the effect of age groups for the other data/variables. Does this mean for the other data/variables, there were no effect of ages?. If the model do not provide significant tests, it is suggested to carry out MANOVA or at least ANOVA to test whether the differences are significant.

- Sensitivity analysis in page 14, line 229-237 should be combined with the similar topics in page 10.

- Add a note below the Table for shortforms that has not been mentioned before. Eg. CI

***Response:*** *The MANOVA analysis was employed to test the differences of age groups and also gender on the dependent variables including change in SSB consumption, change in weight and change in BMI. The results showed a statistically difference between these variables, and has been added to the results. The accordant of sensitivity analysis topic was amended*. *The definition of abbreviations used in the model and entire manuscript were included.*

2. Discussions.

- It is suggested to add implications of the findings.

- Line 248, list the references for the other studies.

***Response:*** *The implication of the findings was discussed more on the importance of tax rates and the impacts of consumption, purchasing behavior and health outcome. Outcomes from a systematic review were added. We also considered the policy situation in the country and found that only SSB tax policy may not be fully working and have stated the importance of multi-sectoral approaches. We also addressed the reference lists of the studies we mentioned in the discussion part.*

3. Need minor proof reading of English.

***Response:*** *This revised manuscript was proofread by an English professional user who has experienced in teaching and editing English documents.*

Reviewer #2: This is yet another modeling study evaluating the impact of SSB tax on obesity. There have been similar modeling studies published in the past. The advantage of this study is that it provides the exercise from a LMIC--Thailand.

However, I have some major and minor concerns as followed.

Major

1. Regarding the actual Thai tax regime, what is the total(calculate) tax rate in each level? These actual tax rate should be used as the based model to explain the real situation of your country. The 20% tax rate as WHO recommended can be used as a scenario to show the discrepancy between the real situation and the recommendation. Moreover, the recent study in Thailand, Markchang et al(2019) showed the evidences of actual SSB price changes and SSB sugar content change. This evidences need to be consider in the model. This might explain the real pass-on rate and the calories from sugar content in SSB as well.

All other scenario which the authors used sensitivity analysis are acceptable to show the uncertainty and compliment the model.

***Response:*** *This study used a percentage increase in SSB price (11%) to reflect the current situation of SSB tax policy in Thailand. The structure of SSB tax is quite complex and the tax rates are different among SSB products. Also, there was no data available for the price elasticity and pass-on rate in Thailand. We, therefore, run a model on a 11% price increase as the result of an imposed tax. This is a main limitation of our model and we suggest the future research to cover this issue.*

2. The SSB tax has already been introduced in Thailand since 2017. This is possible already enough time to change consumption rates. If you can find the recent consumption data after the tax implementation, this might be reasonable and more reliable than assuming on price elasticity.

***Response:*** *The study on the change in SSB consumption after tax was just published in December, 2020. The results showed a 2.5% decrease in SSB consumption (Phulkerd et. Al, 2020). This study has taken this point in the discussion part. However, to assess the price elasticity, some data is needed such as the recent price of SSB. However, the availability of SSB price is in 2017, where it is the first wave of tax implementation. Presently, we are in the second wave of SSB tax policy and there are no studies to estimate change in price of SSB yet.*

1. Please check the accuracy of all your references and revise, i.e. ref#7 and #8 in line 51-54.

***Response:*** *We have adjusted the list of references according to the comment****.***

2. The manuscript is in need of proofreading and correct some typo and grammatical error, i.e. line 79, line 225. Please also make it's consistent between the word "obesity" and "overweight" which one you really mean to.

***Response:*** *We have adjusted the typo and grammatical error, and made a consistency of words according to the comment****.***
